# Metabolic engineering and cultivation strategies for efficient production of fucoxanthin and related carotenoids

**DOI:** 10.1007/s00253-025-13441-1

**Published:** 2025-03-04

**Authors:** Kenya Tanaka, John Chi-Wei Lan, Akihiko Kondo, Tomohisa Hasunuma

**Affiliations:** 1 Engineering Biology Research Center, Kobe University, 1-1 Rokkodai, Nada, Kobe, 657-8501 Japan; 2 Graduate School of Science, Innovation and Technology, Kobe University, 1-1 Rokkodai, Nada, Kobe, 657-8501 Japan; 3 Research Center for Solar Energy Chemistry, Graduate School of Engineering Science, Osaka University, Toyonaka, Osaka 560-8531 Japan; 4 Biorefinery and Bioprocessing Engineering Laboratory, Department of Chemical Engineering and Materials Science, Yuan Ze University, Chungli, Taoyuan 320 Taiwan; 5 Graduate School of Biotechnology and Bioengineering, Yuan Ze University, Chungli, Taoyuan 320 Taiwan; 6 RIKEN Center for Sustainable Resource Science, 1-7-22 Suehiro, Tsurumi, Yokohama, Kanagawa 230-0045 Japan; 7 Department of Chemical Science and Engineering, Graduate School of Engineering, Kobe University, 1-1 Rokkodai, Nada, Kobe, 657-8501 Japan

**Keywords:** Fucoxanthin, Metabolic engineering, Carotenoid biosynthesis, Cultivation optimization

## Abstract

**Abstract:**

Fucoxanthin, a bioactive carotenoid derived from algae, has attracted considerable attention for its applications in health, cosmetics, and nutrition. Advances in metabolic engineering, such as the overexpression of pathway-specific enzymes and enhancement of precursor availability, have shown promising results in improving production efficiency. However, despite its high value, the biosynthetic pathway of fucoxanthin remains only partially elucidated, posing significant challenges for metabolic engineering efforts. Recent studies have identified previously unknown enzymes and regulatory elements within the pathway, providing opportunities for further productivity enhancements through targeted metabolic modifications. Additionally, adaptive evolution, mutagenesis-driven strain development, and optimized cultivation conditions have demonstrated significant potential to boost fucoxanthin yields. This review consolidates the latest insights into the biosynthetic pathway of fucoxanthin and highlights metabolic engineering strategies aimed at enhancing the production of fucoxanthin and related carotenoids, offering approaches to design high-yielding strains. Furthermore, recent advancements in random mutagenesis and cultivation technology are discussed. By integrating these developments, more economically viable and environmentally sustainable fucoxanthin production systems can be achieved.

****Key Points**:**

• *Insights into fucoxanthin biosynthesis enable targeted metabolic engineering.*

• *ALE and cultivation strategies complement metabolic engineering efforts.*

• *Balanced push–pull-block strategies improve fucoxanthin production efficiency.*

## Introduction

Fucoxanthin is a carotenoid pigment predominantly found in algae, particularly in brown macroalgae and certain microalgae. It plays a critical role in facilitating efficient absorption of blue-green light (500 to 580 nm) for photoprotection and light harvesting (Bertrand 2010; Takaichi 2011; Anjana and Arunkumar 2024). Owing to its diverse bioactivities, including antioxidant, anti-obesity, anti-cancer, and anti-diabetic properties, fucoxanthin has garnered substantial interest in the cosmetic, nutraceutical, and pharmaceutical industries (Peng et al. 2011; Christaki et al. 2013; Galasso et al. 2017). Fucoxanthin is predominantly produced from natural sources, as its chemical synthesis has not yet been realized, making its extraction and purification highly resource intensive. Typical methods include harvesting fucoxanthin from brown macroalgae such as *Laminaria* spp. and *Undaria pinnatifida*, as well as microalgae like *Phaeodactylum tricornutum*. These processes often involve energy-intensive cultivation, advanced extraction techniques, and rigorous purification steps. All of them contribute to the high market price of fucoxanthin, underscoring the need for enhanced production efficiency (Pang et al. 2024).

To date, numerous studies have focused on enhancing productivity through cultivation engineering approaches (Wang et al. 2021; Khaw et al. 2022). In biomanufacturing, rational strain engineering using genetic modifications is generally considered effective for increasing productivity (Vavricka et al. 2020; Kato et al. 2022; Tanaka et al. 2024). Nevertheless, gaps remain in the elucidation of fucoxanthin biosynthetic pathways, leaving significant room for improvement in productivity through metabolic engineering approaches. Recent advances have identified fucoxanthin biosynthetic genes in *Phaeodactylum tricornutum* (Dautermann et al. 2020; Bai et al. 2022; Cao et al. 2023). These findings are expected to accelerate the application of metabolic engineering strategies for fucoxanthin production.

**Fig. 1:**
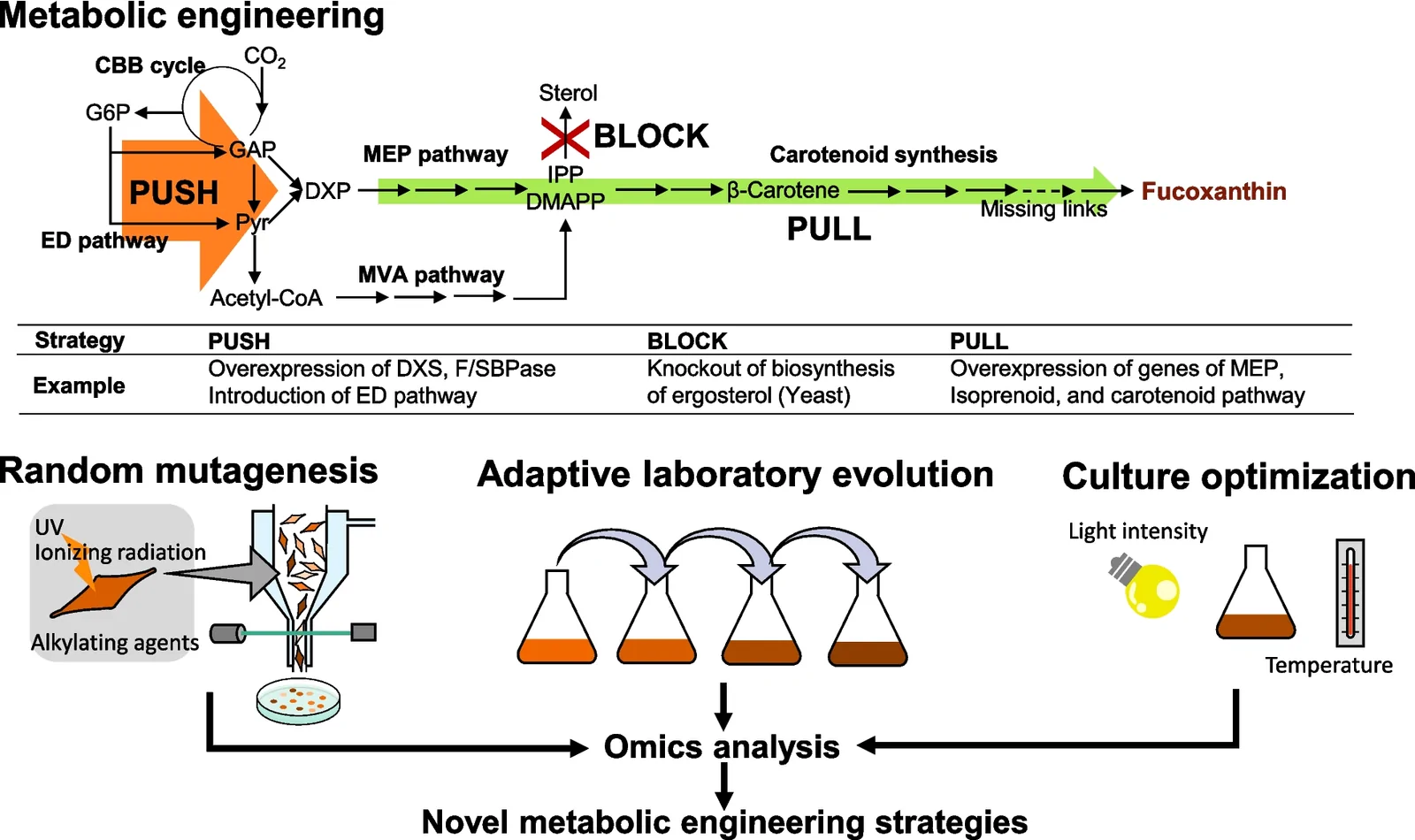
“Push–pull-block” metabolic engineering strategy for developing a fucoxanthin-producing strain. Random mutagenesis, adaptive laboratory evolution, and optimization of culture condition are complementary approaches for fucoxanthin production

This review presents an overview of the current understanding of fucoxanthin biosynthetic pathways and highlights key metabolic engineering strategies that could play a crucial role in enhancing fucoxanthin production (Fig. 1). Additionally, recent advancements in mutation breeding and optimization of cultivation conditions are discussed as complementary approaches to metabolic engineering for improving fucoxanthin yields. Insights gained from omics analyses of mutant strains and various cultivation conditions may further lead to the discovery of novel strategies for metabolic engineering.

## Biosynthetic pathway of fucoxanthin

**Fig. 2:**
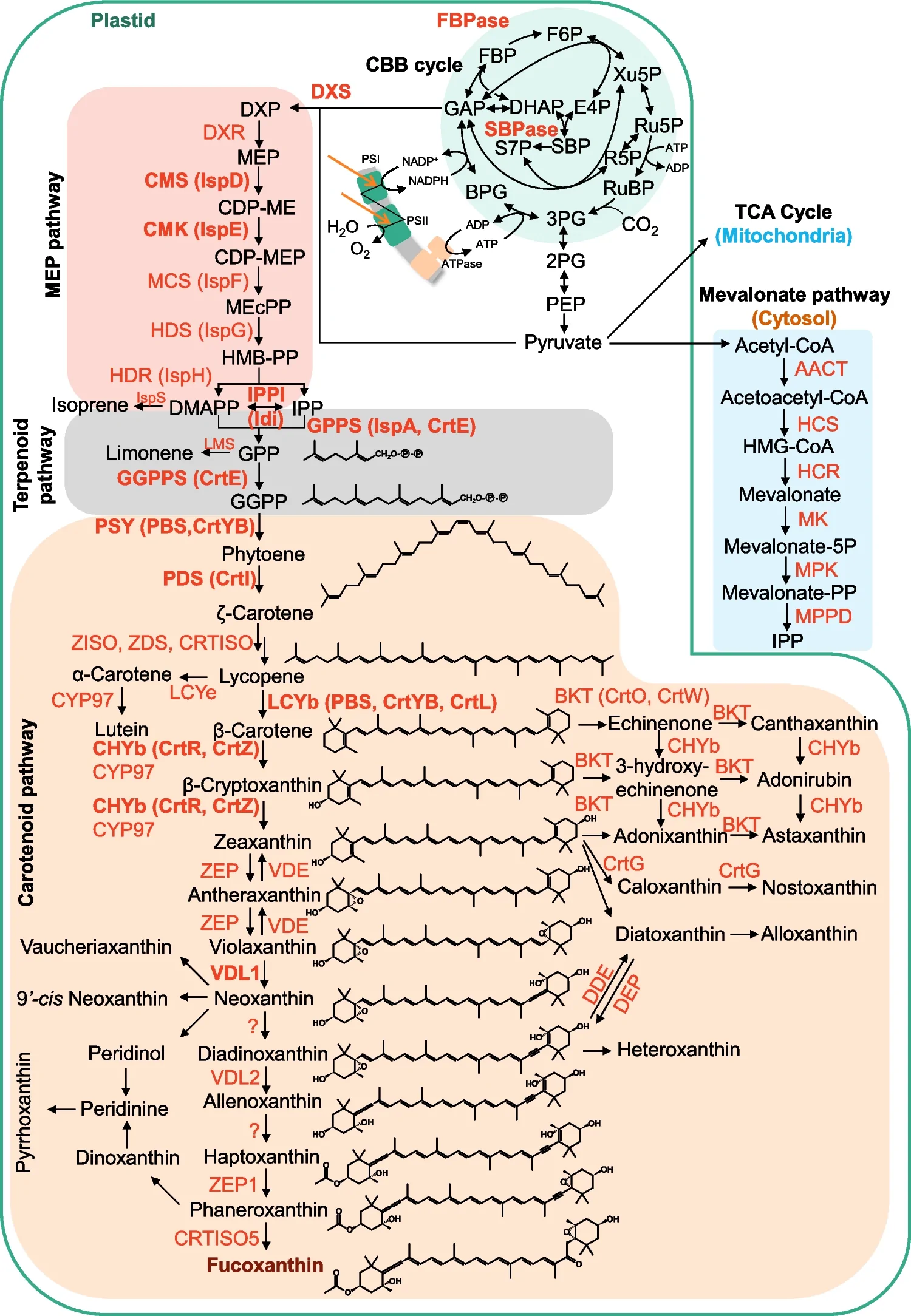
General metabolic pathway of fucoxanthin biosynthesis. Abbreviations: AACT, acetoacetyl-CoA thiolase; BKT, beta-carotenoid ketolase; BPG, 1,3-bisphosphoglycerate; CBB, Calvin–Benson–Bassham; CDP-ME, 4-diphosphocytidyl-2-C-methylerythritol; CDP-MEP, 4-diphosphocytidyl-2-C-methyl-d-erythritol 2-phosphate; CHYb, beta-carotenoid hydroxylase; CMK, 4-diphosphocytidyl-2-C-methyl-d-erythritol kinase; CMS, 2-C-methyl-d-erythritol 4-phosphate cytidylyltransferase; CRTISO, carotenoid isomerase; CYP97, cytochrome P450 hydroxylase; DDE, diadinoxanthin de-epoxidase; DHAP, dihydroxyacetone phosphate; DEP, diatoxanthin epoxidase; DMAPP, dimethylallyl pyrophosphate; DXR, 1-deoxy-d-xylulose 5-phosphate reductoisomerase; DXP, 1-deoxy-d-xylulose 5-phosphate; DXS, 1-deoxy-d-xylulose 5-phosphate synthase; E4P, erythrose 4-phosphate; FBP, fructose 1,6-bisphosphate; F6P, fructose 6-phosphate; GAP, glyceraldehyde 3-phosphate; GGPP, geranylgeranyl diphosphate; GGPPS, geranylgeranyl diphosphate synthase; GPP, geranyl diphosphate; GPPS, geranyl diphosphate synthase; HCR, HMG-CoA reductase; HCS, hydroxymethylglutaryl-CoA synthase; HDR, 4-hydroxy-3-methylbut-2-en-1-yl diphosphate reductase; HDS, 4-hydroxy-3-methylbut-2-en-1-yl diphosphate synthase; HGM-CoA, 3-hydroxy-3-methylglutaryl-CoA; HMB-PP, (E)−4-hydroxy-3-methylbut-2-enyl pyrophosphate; IPP, isopentenyl pyrophosphate; IPPI, isopentenyl-diphosphate isomerase; IspS, isoprene synthase; LCYb, lycopene beta cyclase; LCYe, lycopene epsilon cyclase; MCS, 2-C-methyl-d-erythritol 2,4-cyclodiphosphate synthase; LMS, limonene synthase; MEcPP, 2-C-methyl-d-erythritol 2,4-cyclodiphosphate; MEP, 2-C-methylerythritol 4-phosphate; MK, mevalonate-5-kinase; MPK, phosphomevalonate kinase; MPPD, mevalonate-5-pyrophosphate decarboxylase; NXS, neoxanthin synthase; PDS, phytoene desaturase; PEP, phosphoenolpyruvate; PSI, photosystem I; PSII, photosystem II; PSY, phytoene synthase; 2PG, 2-phosphoglycerate; 3PG, 3-phosphoglycerate; RuBP, ribulose 1,5-bisphosphate; R5P, ribose 5-phosphate; Ru5P, ribulose 5-phosphate; SBP, sedoheptulose 1,7-bisphosphate; S7P, sedoheptulose 7-phosphate; TCA, tricarboxylic acid; VDE, violaxanthin de-epoxidase; VDL, violaxanthin de-epoxidase-like; Xu5P, xylulose 5-phosphate; ZDS, zeta-carotene desaturase; ZEP, zeaxanthin epoxidase; ZISO, zeta-carotene isomerase

The biosynthetic pathway of fucoxanthin, a carotenoid, has not been fully elucidated. To date, candidate genes corresponding to known carotenoid biosynthetic enzymes have been identified through genomic analyses of diatoms, particularly *P. tricornutum* (Bertrand 2010; Dambek et al. 2012). Carotenoid biosynthesis begins with the methylerythritol phosphate (MEP) pathway, which produces dimethylallyl pyrophosphate (DMAPP) and isopentenyl pyrophosphate (IPP) (Fig. 2). These precursors are converted to β-carotene through the sequential actions of phytoene synthase (PSY), phytoene desaturase (PDS), ζ-carotene desaturase (ZDS), and lycopene β-cyclase (LCYb) (Dambek et al. 2012). β-Carotene is subsequently converted to zeaxanthin by β-carotene hydroxylase (CHYb). Zeaxanthin undergoes two epoxidation steps catalyzed by zeaxanthin epoxidase (ZEP) to violaxanthin. Violaxanthin is converted back to zeaxanthin by violaxanthin de-epoxidase (VDE), which is activated by acidification of the thylakoid lumen under high-light conditions in land plants, green algae, and some groups of chromalveolate algae. Together, these reactions constitute the violaxanthin cycle for photoprotective defense (Goss and Jakob 2010). In *P. tricornutum*, the conversion of β-carotene to zeaxanthin is catalyzed by cytochrome P450 enzymes (CYP97) rather than the CHYb (Cui et al. 2019). Among three ZEP genes in *P. tricornutum*, *zep2* likely mediates the conversion of zeaxanthin to violaxanthin (Eilers et al. 2016a; Græsholt et al. 2024).

Fucoxanthin biosynthesis proceeds through neoxanthin, which is derived from violaxanthin (Fig. 2). The enzyme violaxanthin de-epoxidase-like 1 (VDL1), responsible for converting violaxanthin to neoxanthin, was identified in *Nannochloropsis oceanica* using a random insertional mutagenesis screening approach (Dautermann et al. 2020). In *P. tricornutum*, two additional enzymes involved in downstream steps of the pathway have been identified: VDL2, which converts diadinoxanthin to alloxanthin, and ZEP1, which converts haptaxanthin to phaneroxanthin (Bai et al. 2022). In diatoms and haptophytes, including *P. tricornutum*, diadinoxanthin is de-epoxidized to diatoxanthin under high light conditions, where it dissipates excess energy through non-photochemical quenching. Diatoxanthin is epoxidized back into diadinoxanthin under low light conditions, forming diadinoxanthin cycle (Goss et al. 2006). In *P. tricornutum*, diadinoxanthin is de-epoxidized to diatoxanthin by VDE (Lavaud et al. 2012), while diatoxanthin is suggested to be epoxidized back to diadinoxanthin by ZEP3 (Græsholt et al. 2024).

A novel enzyme responsible for the final step of fucoxanthin biosynthesis, CRTISO5, was recently identified. CRTISO5 converts phaneroxanthin to fucoxanthin and, while structurally similar to conventional carotenoid *cis*–*trans* isomerases (CRTISO), exhibits a distinct enzymatic function. Specifically, CRTISO5 catalyzes a hydration reaction at the carbon–carbon triple bond of phaneroxanthin, leading to fucoxanthin production (Cao et al. 2023). In *P. tricornutum* mutants lacking CRTISO5, fucoxanthin synthesis was completely inhibited, and phaneroxanthin accumulated instead, demonstrating the essential role of CRTISO5 in fucoxanthin biosynthesis.

Despite these advancements, the enzymes responsible for the conversion of neoxanthin to diadinoxanthin and alloxanthin to haptaxanthin remain unidentified. Furthermore, fucoxanthin-producing algae, including brown algae (Phaeophytes), golden-brown algae (Chrysophytes), and raphidophyte algae, lack orthologs of CRTISO5 and *P. tricornutum* ZEP1, indicating that they may utilize alternative pathways for fucoxanthin biosynthesis (Bai et al. 2022; Cao et al. 2023).

## Metabolic engineering strategies for fucoxanthin production

Metabolic engineering approaches for efficient fucoxanthin production rely on a detailed understanding of its biosynthetic pathway. Although the complete biosynthetic pathway of fucoxanthin has yet to be fully elucidated, enhancing precursor supply pathways has been suggested as an effective strategy (Table 1). Conversely, substantial progress has been achieved in the metabolic engineering of carotenoids with well-characterized biosynthetic pathways, such as carotenes and astaxanthin, using various genetic engineering techniques (Srivastava et al. 2022; Yu et al. 2024).

**Table 1:** Strain development for carotenoid production using metabolic engineering strategies

Pigment	Species	Strategy	Effect	References
Fucoxanthin	*Phaeodactylum tricornutum*	Introduction of *PSY*	1.45-fold increased production	Kadono et al. 2015
Fucoxanthin	*Phaeodactylum tricornutum*	Expression of *DXS* or *PSY*	24.2 mg/g DCW (DXS), 18.4 mg/g DCW (PSY)	Eilers et al. 2016a, b
Fucoxanthin	*Phaeodactylum tricornutum*	*Vdr/Vde/Zep3* triple overexpression	fourfold increased production	Manfellotto et al. 2020
Fucoxanthin	*Phaeodactylum tricornutum*	Overexpression of *CMK* or *CMS*	1.83-fold (CMK), 1.82-fold (CMS) enhanced production	Hao et al. 2021
Fucoxanthin	*Phaeodactylum tricornutum*	Overexpression of *HSF1*	6.2 mg/g DCW	Song et al. 2023
Fucoxanthin	*Phaeodactylum tricornutum*	Dual overexpression of *DXS* and *LYCB*	6.53 mg/g DCW	Cen et al. 2022
Fucoxanthin	*Phaeodactylum tricornutum*	Overexpression of *VDL1*	Significant increases by 8.2 to 41.7% in fucoxanthin content	Li et al. 2024
Limonene	*Synechocystis* PCC 6803	Expression of limonene synthase, *dxs*, *crtE*, and *ipi*	19 μg/L/day	Kiyota et al. 2014
Isoprene	*Synechocystis* PCC 6803	Expression of *Dxs* and *Ipi*	2.8 mg/g DCW	Englund et al. 2018
β-Carotene	*Chlamydomonas reinhardtii*	Expression of *crtB* gene from *Xanthophyllomyces dendrorhous*	38% enhancement in β-carotene	Rathod et al. 2020
Zeaxanthin	*Synechococcus elongatus* PCC7942	Overexpression of *crtR* gene	1.36-fold increase in yield (mg/g DCW)	Sarnaik et al. 2018
Zeaxanthin	*Chromochloris zofingiensis*	Insertion or substitution in β-carotene ketolase (*BKT*) gene 1	7–11-fold increase (compared to wild type)	Ye and Huang 2019
Canthaxanthin	*Chlamydomonas reinhardtii*	Overexpression of *Cr-bkt* gene	2.34-fold increase in the canthaxanthin	Tran and Kaldenhoff 2020
Astaxanthin	*Xanthophyllomyces dendrorhous*	Introduction of multiple copies of *crtYB*	191% increase compared wild type	Ledetzky et al. 2014
Astaxanthin and canthaxanthin	*Dunaliella salina*	Introduction of *bkt* gene from *Haematococcus pluvialis*	Astaxanthin and canthaxanthin with maximum content of 3.5 and 1.9 μg/g	Anila et al. 2016
Astaxanthin	*Haematococcus pluvialis*	Overexpression of *pds* gene	67% higher astaxanthin content than the wild type	Galarza et al. 2018
Astaxanthin	*Synechocystis* sp. PCC 6803	Expression of *crtW* and *crtZ*	50% increase in astaxanthin accumulation (compared to wild type)	Menin et al. 2019
Astaxanthin	*Synechocystis* sp. PCC 6803	Insertion and expression of *bkt* and *crtR-B* from *H. pluvialis*	4.81 mg/g DCW	Liu et al. 2019
Astaxanthin	*Synechococcus* sp. PCC 7002	Expression of *crtW* and *crtZ*	3 mg/g DCW	Hasunuma et al. 2019
Astaxanthin	*Synechocystis* sp. PCC 6803	Expression of *crtW*, *crtZ*, *F/SBPase*, *dxs*,* ispA*	29.6 mg/g DCW	Diao et al. 2020
Astaxanthin	*Synechocystis* sp. PCC 6803	Expression of *crtW*, *crtZ*, *dxs*, *pds*	1 μg/mL/OD730	Shimada et al. 2020
Astaxanthin	*Chlamydomonas reinhardtii*	*CrBKT* overexpression	4.3 mg/L/day	Perozeni et al. 2020

Several steps of the carotenoid biosynthetic pathways overlap with those involved in fucoxanthin synthesis (Fig. 2). Consequently, the metabolic engineering strategies established for these carotenoids could be adapted for engineering strains to enhance fucoxanthin production. A fundamental approach in metabolic engineering is the push–pull-block strategy (Fig. 1). In the context of fucoxanthin production, the push strategy aims to increasing precursor availability by enhancing the methylerythritol phosphate (MEP) pathway, while the pull strategy focuses on upregulating downstream pathways involved in carotenoid biosynthesis. The block strategy involves knocking out or downregulating competing pathways, such as those involved in the synthesis of other isoprenoids. Employing a balanced combination of these strategies can lead to substantial improvements in carotenoid production yields (Lyu et al. 2022). The enzymes involved in the fucoxanthin biosynthetic pathway in *P. tricornutum*, as well as the enzymes introduced through metabolic engineering approaches, are summarized in Table 2. The following sections outline specific strategies organized by each segment of the pathway.

**Table 2:** List of enzymes related to fucoxanthin biosynthesis in *Phaeodactylum tricornutum* and/or utilized in metabolic engineering for fucoxanthin production

Enzyme name	Symbol	Organism	UniProt or GenBank	Function	Reference
Fructose-1,6-/sedoheptulose 1,7-bisphosphatase	FBP/SBPase	*Synechococcus* sp. PCC7002	B1XLK5	FBP > F6P, SBP > S7P	Diao et al. 2020
1-Deoxy-d-xylulose 5-phosphate synthase	DXS	*Phaeodactylum tricornutum*	B7S452	GAP + Pyr > DXP	Eilers et al. 2016a, b, Diao et al. 2020
1-Deoxy-d-xylulose 5-phosphate synthase	DXS	*Synechocystis* sp. PCC 6803	sll1945	GAP + Pyr > DXP	Kiyota et al. 2014
1-Deoxy-d-xylulose-5-phosphate reductoisomerase	DXR	*Phaeodactylum tricornutum*	B7FQZ5	DXP > MEP	
2-C-Methyl-d-erythritol 4-phosphate cytidylyltransferase	CMS (IspD)	*Phaeodactylum tricornutum*	B7G4H5	MEP > CDP-ME	
4-(Cytidine 5′-diphospho)−2-C-methyl-d-erythritol kinase	CMK (IspE)	*Phaeodactylum tricornutum*	B7FUR0	CDP-ME > CDP-MEP	Hao et al. 2021
2-C-Methyl-d-erythritol 2,4-cyclodiphosphate synthase	MCS (IspF)	*Phaeodactylum tricornutum*	B7FYU1, B7FYU2	CDP-MEP > MEcPP	Hao et al. 2021
1-Hydroxy-2-methyl-2-(E)-butenyl-4-diphosphate synthase	HDS (IspG)	*Phaeodactylum tricornutum*	B7FV10	MEcPP > HMB-PP	
4-Hydroxy-3-methylbut-2-enyl diphosphate reductase	HDR (IspH)	*Phaeodactylum tricornutum*	B7FUL0	HMB-PP > DMAPP, HMB-PP > IPP	
Isopentenyl pyrophosphate isomerase	Ipi	*Synechocystis* sp. PCC 6803	P74287	DMAPP = IPP	Kiyota et al. 2014, Englund et al. 2018
Farnesyl diphosphate synthase	FPPS	*Phaeodactylum tricornutum*	B7GA81	GPP + IPP > FPP	
Farnesyl diphosphate synthase	IspA	*Escherichia coli*	P22939	GPP + IPP > FPP	Diao et al. 2020
Geranylgeranyl diphosphate synthase	CrtE	*Synechocystis* sp. PCC 6803	P72683	DMAPP + IPP > GPP, GPP + IPP > FPP, FPP + IPP > GGPP	Kiyota et al. 2014, Satta et al. 2022
Geranylgeranyl diphosphate synthase	GGPPS	*Phaeodactylum tricornutum*	B7G3T2, B7FU89	FPP + IPP > GGPP	
15-*cis*-Phytoene synthase	PSY (PBS)	*Phaeodactylum tricornutum*	B7FVW3	GGPP > phytoene	Kadono et al. 2015, Eilers et al. 2016a, b
Bifunctional lycopene cyclase/phytoene synthase	CrtYB	*Xanthophyllomyces dendrorhous*	Q7Z859	GGPP > phytoene, lycopene > β-carotene	Ledetzky et al. 2014, Rathod et al. 2020
Phytoene desaturase	PDS (CrtI)	*Haematococcus pluvialis*	O65813	Phytoene > ζ-carotene	Galarza et al. 2018
Phytoene desaturase	PDS (CrtI)	*Synechocystis* sp. PCC 6803	P29273	Phytoene > ζ-carotene	Shimada et al. 2020
Phytoene desaturase	PDS (CrtI)	*Phaeodactylum tricornutum*	B5Y4Q5	Phytoene > ζ-carotene	Dambek et al. 2012
ζ-Carotene desaturase	ZDS	*Phaeodactylum tricornutum*	B7FPC4	ζ-Carotene > prolycopene	
Carotenoid isomerase	CRTISO1	*Phaeodactylum tricornutum*	B7FXV4		
Carotenoid isomerase	CRTISO2	*Phaeodactylum tricornutum*	B7G5L7		
Carotenoid isomerase	CRTISO3	*Phaeodactylum tricornutum*	B7G5U6		
Carotenoid isomerase	CRTISO4	*Phaeodactylum tricornutum*	B7FWY8	Prolycopene > lycopene	Sun et al. 2022
Carotenoid isomerase	CRTISO5	*Phaeodactylum tricornutum*	B7FQF7	Phaneroxanthin > fucoxanthin	Cao et al. 2023
Lycopene beta-cyclase	LCYB	*Phaeodactylum tricornutum*	B7FNX5	Lycopene > β-carotene	
Cytochrome P450 beta hydroxylase	CYP97A	*Phaeodactylum tricornutum*	A0A3S7L8P2	β-Carotene > zeaxanthin	Cui et al. 2019
β-Carotene oxygenase	CrtR	*Synechococcus* PCC 7002	B1XIX7	β-Carotene > zeaxanthin	Sarnaik et al. 2018
Carotenoid hydroxylase	crtR-B	*Haematococcus pluvialis*	AF162276.1	β-Carotene > zeaxanthin	Liu et al. 2019
β-Carotene hydroxylase	CrtZ	*Brevundimonas* sp. SD-212	MK214313	β-Carotene > zeaxanthin	Menin et al. 2019
4,4′β-Carotene oxygenase	CrtW	*Brevundimonas* sp. SD-212	MK214312	Zeaxanthin > astaxanthin	Menin et al. 2019
β-Carotene ketolase	BKT	*Haematococcus pluvialis*	AY603347.1	Zeaxanthin > astaxanthin	Liu et al. 2019
β-Carotene ketolase	BKT	*Chlamydomonas reinhardtii*	Q4VKB4	Zeaxanthin > astaxanthin	Perozeni et al. 2020, Tran and Kaldenhoff 2020
Zeaxanthin epoxidase	ZEP1	*Phaeodactylum tricornutum*	B7FYW4	Haptoxanthin > phaneroxanthin	Bai et al. 2022
Zeaxanthin epoxidase	ZEP2	*Phaeodactylum tricornutum*	B7FQV6	Zeaxanthin > violaxanthin	Eilers et al. 2016a, Græsholt et al. 2024
Zeaxanthin epoxidase	ZEP3	*Phaeodactylum tricornutum*	B7FUR7	Diatoxanthin > diadinoxanthin	Manfellotto et al. 2020, Græsholt et al. 2024
Violaxanthin de-epoxidase	VDE	*Phaeodactylum tricornutum*	B7FUR6	Violaxanthin > zeaxanthin	Manfellotto et al. 2020
Violaxanthin de-epoxidase-like	VDL1	*Phaeodactylum tricornutum*	B7G087	Violaxanthin > neoxanthin	Dautermann et al. 2020, Li et al. 2024
Violaxanthin de-epoxidase-like	VDL2	*Phaeodactylum tricornutum*	B7FYW5	Diadinoxanthin > allenoxanthin	Bai et al. 2022
Violaxanthin de-epoxidase-related	VDR	*Phaeodactylum tricornutum*	B7FR37		Manfellotto et al. 2020

## Enhancement of the MEP pathway

Enhancing the MEP pathway, which produces IPP, a common precursor of carotenoids, has been demonstrated to be effective for increasing the production of many carotenoids, including fucoxanthin. In *P. tricornutum*, overexpression of the gene encoding 1-deoxy-d-xylulose 5-phosphate synthase (DXS), which catalyzes the first step of the MEP pathway, resulted in a 2.4-fold increase in fucoxanthin content compared to the wild-type strain (Eilers et al. 2016b). In addition to *DXS*, the overexpression of *LCYB* achieved production levels of 6.53 and 4.34 mg/g DCW for fucoxanthin and β-carotene, respectively (Cen et al. 2022). Overexpression of *DXS* has been widely employed to enhance the production of various terpenoids and carotenoids, such as limonene, isoprene, and astaxanthin, with its efficacy well documented (Kiyota et al. 2014; Englund et al. 2018; Diao et al. 2020; Shimada et al. 2020). Furthermore, overexpression of 4-diphosphocytidyl-2-C-methyl-d-erythritol kinase (CMK) and 2-C-methyl-d-erythritol 4-phosphate cytidylyltransferase (CMS) genes in *P. tricornutum*, which participate in subsequent steps of the MEP pathway, has also been shown to increase fucoxanthin accumulation by 83 and 82%, respectively (Hao et al. 2021).

## Enhancement of the terpenoid biosynthetic pathway

Terpenoid biosynthesis begins with IPP and DMAPP derived from the MEP pathway (Fig. 1). Overexpression of *ipi* (isopentenyl pyrophosphate isomerase) and *crtE* (geranyl pyrophosphate synthase) has been shown to effectively increase the production of isoprenoids. Overexpression of *crtE* and *ipi* together with *dxs* in the limonene-producing strain resulted in a 37% increase in limonene titer in *Synechocystis* sp. PCC 6803 (Kiyota et al. 2014). Overexpressing *ipi* in *Synechocystis* also gave 1.9-fold increase in isoprene production (Englund et al. 2018). Additionally, *ispA* (farnesyl diphosphate synthase) overexpression has been reported to enhance astaxanthin production (Diao et al. 2020). Given these findings, it is plausible that enhancing gene expression of terpenoid synthesis could also contribute to increased production of fucoxanthin as well as other carotenoids.

## Enhancement of the carotenoid biosynthetic pathway

Carotenoid biosynthesis begins with the conversion of geranylgeranyl pyrophosphate (GGPP) into phytoene by PSY, followed by the production of ζ-carotene from phytoene by PDS. Overexpression of these key enzymes in the carotenoid biosynthetic pathway has been shown to enhance fucoxanthin production in *P. tricornutum.* Specifically, introducing *PYS* under the control of *fcpA* promoter increased fucoxanthin content 1.45-fold compared to the levels in the wild-type strain (Kadono et al. 2015). Similarly, *PSY* introduction resulted in a 1.8-fold higher fucoxanthin content relative to wild type (Eilers et al. 2016b). Overexpression of PDS in the chloroplast of *Haematococcus pluvialis* showed a 67% increase in astaxanthin content compared to the wild type (Galarza et al. 2018). The effectiveness of *PDS* overexpression has also been demonstrated in *Synechocystis* sp. PCC 6803 for astaxanthin production (Shimada et al. 2020), highlighting its potential utility as a target for pathway enhancement across various host systems.

Lycopene, a critical intermediate in the carotenoid biosynthetic pathway, serves as a precursor for several downstream carotenoids. Lycopene is converted into α-carotene by lycopene epsilon cyclase (LCYe) and into β-carotene by LCYb. Since β-carotene is the precursor for both astaxanthin and fucoxanthin, enhancing LCYb activity is crucial for boosting their production. The *crtYB* gene, which encodes a bifunctional enzyme with PSY and LCYb activities, has been identified as a key target for pathway optimization (Verdoes et al. 2003). Introducing *crtYB* in *Xanthophyllomyces dendrorhous* resulted in a 191% increase in astaxanthin content compared to the wild-type strain (Ledetzky et al. 2014).

The introduction of β-carotene hydroxylase genes, such as *crtR* or *crtZ*, has been shown to enhance carotenoid production. For example, introducing *crtR* from *Synechococcus* sp. PCC 7002 into *Synechococcus* sp. PCC 7942 increased zeaxanthin yield by 1.36-fold compared to the wild-type strain (Sarnaik et al., 2017). Similarly, *crtZ* has been reported to contribute to increased carotenoid productivity in other host systems (Liu et al. 2019). In the fucoxanthin biosynthetic pathway after violaxanthin, overexpression of *VDL1* increased fucoxanthin content by 8.2 to 41.7% without negatively affecting growth (Li et al. 2024). Future studies are needed to elucidate the effects of overexpressing other enzymes, such as VDL2, ZEP1, and CRTISO5, on fucoxanthin production.

## Other engineering targets

Productivity can also be improved by modifying enzymes or regulatory factors of the Calvin cycle. Overexpression of fructose-1,6/sedoheptulose-1,7-bisphosphatase (F/SBPase) gene, a key rate-limiting enzyme in the Calvin cycle, has been reported to enhance astaxanthin content by 27% (Diao et al. 2020). Glyceraldehyde-3-phosphate (GAP) and pyruvate, which are the initial substrates of the methylerythritol phosphate (MEP) pathway, can be generated through the Entner–Doudoroff (ED) pathway. Supplying pyruvate and GAP via the ED pathway has been reported to effectively enhance the production of MEP pathway–derived compounds (Liu et al. 2013, 2014; Li et al. 2015). In *P. tricornutum*, overexpression of *HSF1*, a heat shock transcription factor that responds to various stresses such as nutrient deprivation, resulted in an increase in fucoxanthin content by 64 to 99% (Song et al. 2023). HSF1 has been suggested to positively regulate DXS, a key enzyme in the MEP pathway.

## Random mutagenesis and adaptive laboratory evolution

Random mutagenesis using appropriate mutagens or adaptive laboratory evolution (ALE) can effectively enhance carotenoid production, including fucoxanthin (Bleisch et al. 2022; Trovao et al. 2022). Yi et al. combined UV-C mutagenesis with adaptive evolution in *P. tricornutum*, leading to improved fucoxanthin productivity (Yi et al. 2015). Following UV treatment, mutant strains with 1.7-fold higher fucoxanthin content compared to the wild type were obtained. Adaptive evolution further enhanced tolerance to photooxidative stress and improved light-harvesting efficiency. In subsequent studies, the same group employed chemical mutagens such as ethyl methanesulfonate (EMS) and *N*-methyl-*N*′-nitro-*N*-nitrosoguanidine (NTG) combined with fluorescence-based high-throughput screening to select *P. tricornutum* mutants (Yi et al. 2018). This approach yielded mutant strains with up to 69.3% higher fucoxanthin content. Wang et al. (2023a, b) developed a mixotrophic *Nitzschia closterium* strain using glucose as a carbon source through ALE. This strain exhibited enhanced carbon metabolism, resulting in a 79.2% increase in fucoxanthin productivity (Wang et al. 2023a, b). In the resulting strain, carbon flux toward the TCA cycle and the levels of sugar phosphates were enhanced, providing sufficient ATP and NADPH. However, identifying specific causal genes through random mutagenesis and ALE remains challenging. To further understand the genetic basis, whole-genome sequencing of the evolved strain would be required.

## Culture conditions for fucoxanthin production and adaptation mechanisms

Besides metabolic engineering strategy, optimizing culture conditions is a critical strategy for significantly enhancing fucoxanthin production efficiency. Numerous studies have reported the effects of various culture parameters on fucoxanthin production, which have been summarized in several reviews (Wang et al. 2021; Khaw et al. 2022).

Among model organisms, *P. tricornutum* has been extensively studied for its capability to produce fucoxanthin (Pang et al. 2024). Under high light conditions (300 μmol photons m^−2^ s^−1^), the expression of many carotenoid biosynthetic genes is downregulated, and fucoxanthin content decreases significantly (Ding et al. 2023). When shifted to low light conditions (50 μmol photons m^−2^ s^−1^), the expression of some genes recovers including genes encoding light-harvesting complexes, and fucoxanthin content returns to its original level (Ding et al. 2023). Among the recovered genes, GGPPS, a key enzyme in carotenoid biosynthesis, is likely to contribute to the fucoxanthin recovery. Similarly, in *Isochrysis galbana*, high light conditions (300 μmol photons m^−2^ s^−1^) lead to reduced fucoxanthin content and productivity (Li et al. 2022). This reduction is believed to be caused by the downregulation of MEP pathway genes. In addition to light intensity, the wavelength of light also influences fucoxanthin production. In *I. galbana*, green light has been shown to activate genes related to photosynthetic antenna proteins and carotenoid biosynthesis likely via MYB family transcription factors, thereby increasing fucoxanthin production (Chen et al. 2023).

Recently, the haptophyte *Pavlova* sp. has garnered attention as a promising strain for commercial production due to its lack of a cell wall, which facilitates easier extraction of fucoxanthin. Compared to other brown marine microalgae such as *Skeletonema costatum* and *Chaetoceros gracilis*, *Pavlova* sp. exhibits a higher capacity for fucoxanthin production (Chen et al. 2023). Kanamoto et al. conducted a series of developments, including strain selection, optimization of culture conditions, and scale-up studies for *Pavlova* sp. They achieved a fucoxanthin productivity of 4.88 mg/L/day under outdoor cultivation using the *Pavlova* sp. OPMS 30543 strain in an acrylic pipe photobioreactor with 60-mm diameter (Kanamoto et al. 2021). Their finding revealed that fucoxanthin production was higher when 400 mg/L NaNO_3_ was used as the nitrogen source compared to NH_4_Cl. Metabolomic analysis further demonstrated that the presence of NaNO_3_ increased the levels of intermediate metabolites related to fucoxanthin biosynthesis, such as 2-C-methyl-d-erythritol 2,4-cyclodiphosphate (MEcPP), β-carotene, and diadinoxanthin (Yoshida et al. 2023). In *Pavlova* sp. and other algae, mixotrophic cultivation using organic carbon sources such as glycerol has proven effective, enhancing metabolic activity and increasing fucoxanthin productivity. In the *Pavlova gyrans* OPMS 30543X strain, the highest fucoxanthin production was achieved under mixotrophic conditions with 10 mM glycerol and a light intensity of 100 μmol photons m^−2^ s^−1^ (Yoshida et al. 2024). In *Cylindrotheca* sp., the addition of glycerol (2 g/L) was reported to increase fucoxanthin production by 29% (Wang et al. 2023a, b). Glycerol is converted into GAP, one of the starting substrates of the MEP pathway, through the actions of glycerol kinase, glycerol-3-phosphate dehydrogenase, and triose-phosphate isomerase. Therefore, it may be effective for enhancing the production of carotenoids, including fucoxanthin. Conversely, glucose supplementation (5 g/L) in *Nitzschia laevis* enhances the yield of eicosapentaenoic acid (EPA), while simultaneously decreasing fucoxanthin content, suggesting a shift in metabolic priorities (Mao et al. 2021). The reduction in fucoxanthin production is likely associated with the decreased gene expression of key enzymes in the carotenoid biosynthetic pathway, specifically PDS and ZISO.

## Challenges and future prospects in metabolic engineering for fucoxanthin production

The biosynthetic pathway of fucoxanthin involves numerous enzymatic reactions, yet the identification of rate-limiting steps (bottlenecks) remains incomplete. Metabolomics has been proposed as an effective tool for identifying such bottlenecks (Vavricka et al. 2020; Kato et al. 2022). Additionally, integrating machine learning with metabolomics facilitates the identification of key gene targets (Tanaka et al. 2024). In pathways characterized by complex regulatory mechanisms, such as the MEP pathway, a thorough understanding of these regulatory processes is crucial (Volke et al. 2019). For instance, in *P. tricornutum*, the DXS enzyme is regulated at the transcriptional level by the heat shock transcription factor HSF1 (Song et al. 2023). Carotenoid biosynthetic genes are also significantly influenced by light intensity and wavelength through transcription factors such as those of the MYB family proteins (Li et al. 2022; Chen et al. 2023). Modulating the expression levels of these transcription factors could broadly impact the expression of carotenoid biosynthetic genes, leading to significant improvements in fucoxanthin production. These gene expression changes have been revealed through transcriptome analysis, demonstrating that analyzing the effects of different cultivation conditions on fucoxanthin production could facilitate the development of novel metabolic engineering approaches (Fig. 1).

The introduction of engineered enzymes is an effective strategy for enhancing carotenoid production. For example, CrtZ variants engineered to improve astaxanthin production may also be applicable to fucoxanthin biosynthesis. In *Escherichia coli*, the fusion of *Pantoea agglomerans* CrtZ with the glycerol channel protein GlpF for membrane localization enhanced astaxanthin production (Ye et al. 2018). Similarly, CrtZ from *Brevundimonas* sp. SD212, fused via a hydrophilic linker, increased astaxanthin production by 1.4-fold in *E. coli* (Nogueira et al. 2019). Lycopene production has also been enhanced through the directed evolution of *Xanthophyllomyces dendrorhous* CrtE and *P. agglomerans* CrtB (Hong et al. 2019). The activity of PSY, a key rate-limiting enzyme in carotenoid biosynthesis, is highly sensitive to even slight modifications in its amino acid sequence (Zhou et al. 2022). Since the effectiveness of enzyme engineering for fucoxanthin production has not yet been demonstrated, this approach holds great potential for improving fucoxanthin productivity.

Heterologous hosts such as *E. coli*, *S. cerevisiae*, and cyanobacteria are fast growing, genetically tractable, and suitable for fermentation-based production. However, successful fucoxanthin production in these systems requires the complete elucidation of its biosynthetic pathway. For carotenoids with well-characterized pathways, such as astaxanthin, metabolic engineering has been extensively applied in *Yarrowia lipolytica* and *S. cerevisiae* (Yu et al. 2024). Violaxanthin, a precursor of fucoxanthin, can be produced in *S. cerevisiae* (Cataldo et al. 2020). In these organisms, IPP is supplied through the mevalonate (MVA) pathway, where engineering efforts have focused on strengthening acetyl-CoA supply and overexpressing MVA pathway genes, such as 3-hydroxy-3-methylglutaryl coenzyme-A (HMG-CoA) reductase. In the case of yeast carotenoid production, the knockout of the ergosterol biosynthetic gene CYP61 increased astaxanthin titer, indicating that the “block” strategy is also effective for carotenoid production (Yamamoto et al. 2016). Resolving the missing links in the fucoxanthin biosynthetic pathway will likely enable high-level production in heterologous hosts, paving the way for industrial-scale applications in the future.

## Data Availability

All data included in this study are available upon request by contact with the corresponding author.
